# Supplementary figures and images for: COVID-19 Vaccine Effectiveness at a Referral Hospital in Northern Peru: A Retrospective Cohort Study
Source: Vaccines (Basel). 2022 May 20;10(5):812. doi: 10.3390/vaccines10050812 (PMC9143947; doi:10.3390/vaccines10050812)

28-day survival in hospitalized patients for COVID-19

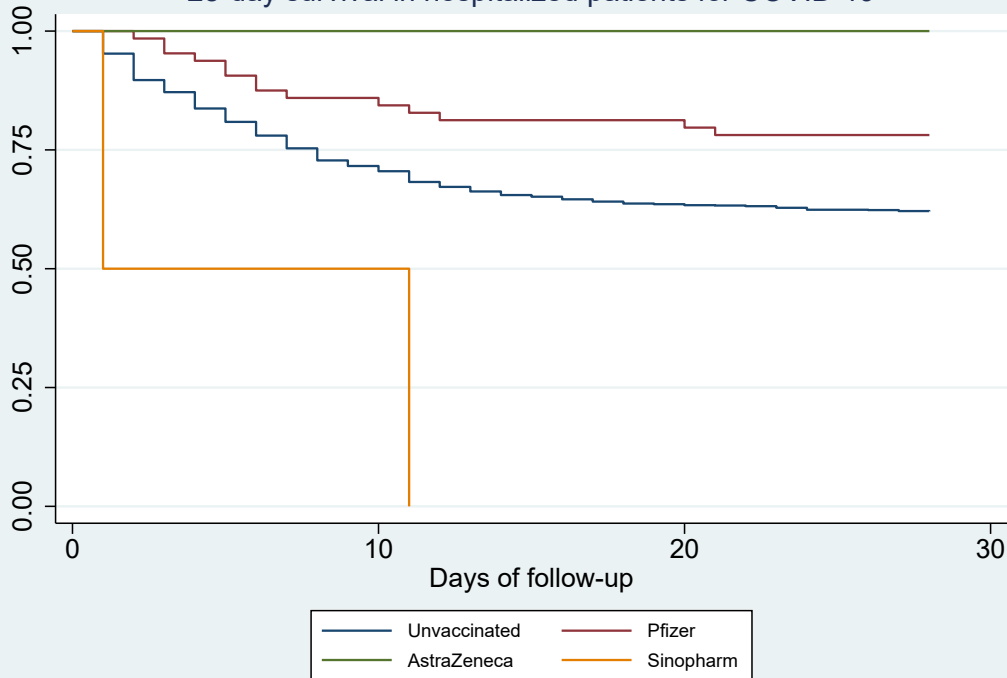

Supplement: Supplementary file 1 [file vaccines-10-00812-s001.zip › Figure_S1_28 day mortality by vaccine type.pdf]

Survival at three months in hospitalized patients for COVID-19

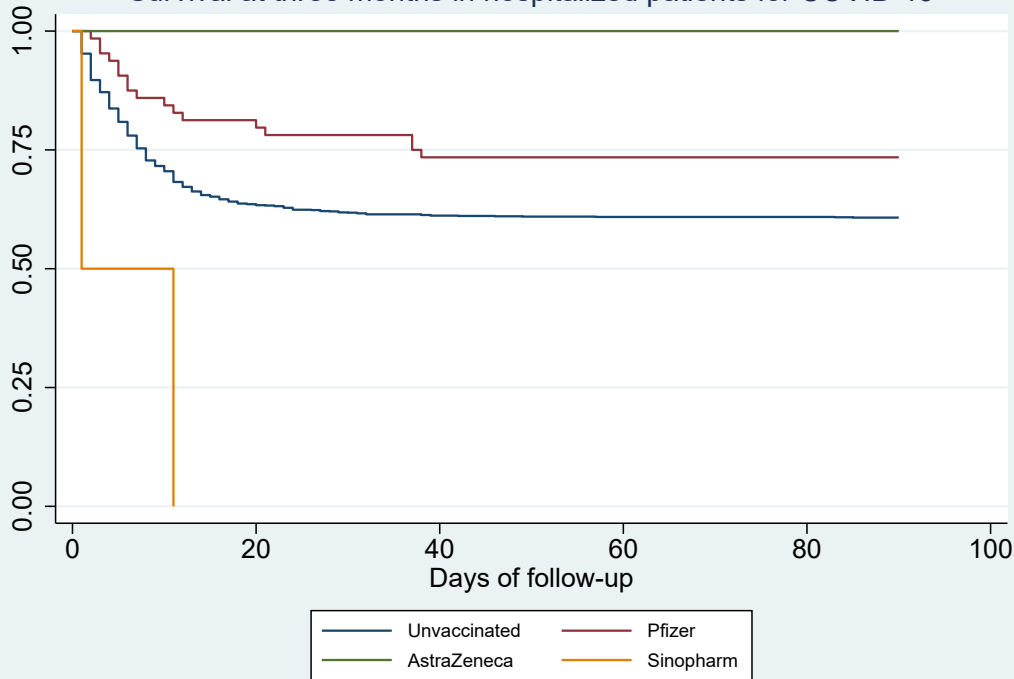

Supplement: Supplementary file 1 [file vaccines-10-00812-s001.zip › Figure_S2_3 month mortality by vaccine type.pdf]
